# Dissecting the Genetic Architecture of Biofuel-Related Traits in a Sorghum Breeding Population

**DOI:** 10.1534/g3.120.401582

**Published:** 2020-12-01

**Authors:** Motoyuki Ishimori, Hideki Takanashi, Kosuke Hamazaki, Yamato Atagi, Hiromi Kajiya-Kanegae, Masaru Fujimoto, Junichi Yoneda, Tsuyoshi Tokunaga, Nobuhiro Tsutsumi, Hiroyoshi Iwata

**Affiliations:** * Department of Agricultural and Environmental Biology, Graduate School of Agricultural and Life Sciences, The University of Tokyo, Bunkyo, Tokyo, Japan, 113-8657; † EARTHNOTE Co. Ltd., Ginoza, Okinawa, Japan, 904-1303

**Keywords:** sorghum, genomic prediction, Bayesian alphabet, breeding population, GWAS, GenPred, Shared data resources

## Abstract

In sorghum [*Sorghum bicolor* (L.) Moench], hybrid cultivars for the biofuel industry are desired. Along with selection based on testcross performance, evaluation of the breeding population *per se* is also important for the success of hybrid breeding. In addition to additive genetic effects, non-additive (*i.e.*, dominance and epistatic) effects are expected to contribute to the performance of early generations. Unfortunately, studies on early generations in sorghum breeding programs are limited. In this study, we analyzed a breeding population for bioenergy sorghum, which was previously developed based on testcross performance, to compare genomic selection models both trained on and evaluated for the *per se* performance of the 3^rd^ generation S_0_ individuals. Of over 200 ancestral inbred accessions in the base population, only 13 founders contributed to the 3^rd^ generation as progenitors. Compared to the founders, the performances of the population *per se* were improved for target traits. The total genetic variance within the S_0_ generation progenies themselves for all traits was mainly additive, although non-additive variances contributed to each trait to some extent. For genomic selection, linear regression models explicitly considering all genetic components showed a higher predictive ability than other linear and non-linear models. Although the number and effect distribution of underlying loci was different among the traits, the influence of priors for marker effects was relatively small. These results indicate the importance of considering non-additive effects for dissecting the genetic architecture of early breeding generations and predicting the performance *per se*.

Sorghum [*Sorghum bicolor* (L.) Moench] is a promising bioenergy crop (Regassa and Wortmann 2014). Commercial F_1_ hybrid sorghums for biofuel production need to exhibit superiority in multiple traits, such as biomass, sugar content, and stress tolerance. In hybrid breeding, testcrosses are generally used to evaluate progeny performance. Many studies have focused on the relationship between the performance of partially or completely inbred lines and their testcrosses to a common unrelated tester to predict the effectiveness of selection on line *per se* performance for improving testcross performance. In maize, the correlation between line *per se* and testcross performance was intermediate to high for some traits, but small for grain yield (Mihaljevic *et al.* 2005). Similarly, other studies in maize and rye revealed that the correlations between phenotypes measured in lines *per se* and their testcrosses were often small for complex traits (Bekavac *et al.* 2008; Falke *et al.* 2010; Miedaner *et al.* 2014).

Although the breeding value of lines or individual breeding candidates for testcross performance is the most important selection criterion in hybrid breeding, the characteristics of the *per se* are also considered because they impact the efficiency of F_1_ seed production. For example, dwarf genotypes, which are easier to handle and more resistant to lodging than taller ones, are often utilized as the seed parents in hybrid sorghum (Pedersen *et al.* 2013). Cytoplasmic male sterility (CMS) is also necessary for hybrid seed parents because sorghum is a predominantly self-pollinated crop (Rooney 2007). Conversely, the pollen parent needs the complementary phenotypes, in which multiple traits (*e.g.*, culm length, panicle length, and heading days) are suitable for hybridization. In a sorghum hybrid breeding program, only candidates with the suitability as hybrid parents are preselected in the advanced generations (*e.g.*, F_5_ lines) before the evaluation based on testcross (Rooney 2007). On the other hand, for utilizing testcrosses for selection beginning in early generations, the evaluation of the breeding population *per se* is appropriately incorporated into the breeding process.

In addition to the additive genetic effects for parents, the performance of early generations *per se* can be affected by non-additive effects, especially dominance due to high heterozygosity before inbreeding has advanced. In sorghum, biomass-related traits (*e.g.*, plant height) showed a considerable level of dominance while others were primarily additive (Felderhoff *et al.* 2012). In addition to additive and dominance effects, epistasis also contributed to various traits for bioenergy sorghum (Shiringani *et al.* 2010; Shiringani and Friedt 2011). The degree of non-additive effects has been examined in various traits across species (Yu *et al.* 1997; Lu *et al.* 2003; Frascaroli *et al.* 2007; Jiang *et al.* 2017). However, the contribution of non-additive effects in early generations of inbreeding in populations derived from several selection cycles is poorly understood. In contrast to genetic mapping populations (*e.g.*, F_2_), breeding populations also have other complications (*e.g.*, unequal allele frequencies) that need to be considered in dissecting the genetic architecture (Würschum 2012). Therefore, statistical models considering the genetic properties of breeding populations with limited inbreeding are necessary.

Genomic prediction (GP) was proposed to evaluate genetic potentials by the regression of target traits on genome-wide dense markers (Meuwissen *et al.* 2001). In plant and animal breeding, GP models have primarily been based on additive effects although non-parametric regression models considering non-additive effects are also used (Hayes and Goddard 2010; Jannink *et al.* 2010). Recently, the importance of non-additive effects in GP was considered (Varona *et al.* 2018). Some studies showed that GP models explicitly including non-additive effects improved prediction accuracy (Su *et al.* 2012; Nishio and Satoh 2014; Jiang and Reif 2015; Vitezica *et al.* 2017). The use of GP models accounting for only additive effects is not suitable when genetic architecture is predominantly regulated by non-additive effects (Howard *et al.* 2014). Alves *et al.* (2019) suggested that Bayesian GP models have the advantage of dissecting complex genetic architecture regulated by non-additive effects.

Bayesian GP models can be applied for a genome-wide association study (GWAS) (Fernando and Garrick 2013). In Bayesian GP models, different priors for marker effects, *e.g.*, Bayesian ridge regression (BRR) and BayesA, are utilized for dealing with various genetic architectures of traits (reviewed by Gianola 2013). In particular, the number of QTL is an important factor in addition to the number of independent chromosome segments for selecting the appropriate prior (Daetwyler *et al.* 2010). For example, Wolc *et al.* (2016) showed that BayesB was suitable in the presence of QTL with large effects. The suitability of priors depends on multiple factors (*e.g.*, heritability, marker density, and the training dataset size), and therefore the optimal settings for prior are generally unknown (de los Campos *et al.* 2013).

The objective of this study is to give insights into the genetics of important agronomic performance *per se* in a non-inbred (S_0_) generation of a bioenergy sorghum population that was selected based on testcross performance. The primary objectives of this research are: i) description of the genetic architecture of the S_0_ generation *per se* performance, ii) utilization of GP for evaluating the performance of the breeding population *per se*, and iii) comparison among models and priors for target traits. Finally, we discuss the importance and reliability of modeling non-additive effects for non-inbred and early inbreeding generations in plant breeding programs.

## Materials and Methods

### Mating design of breeding population

**Figure 1:**
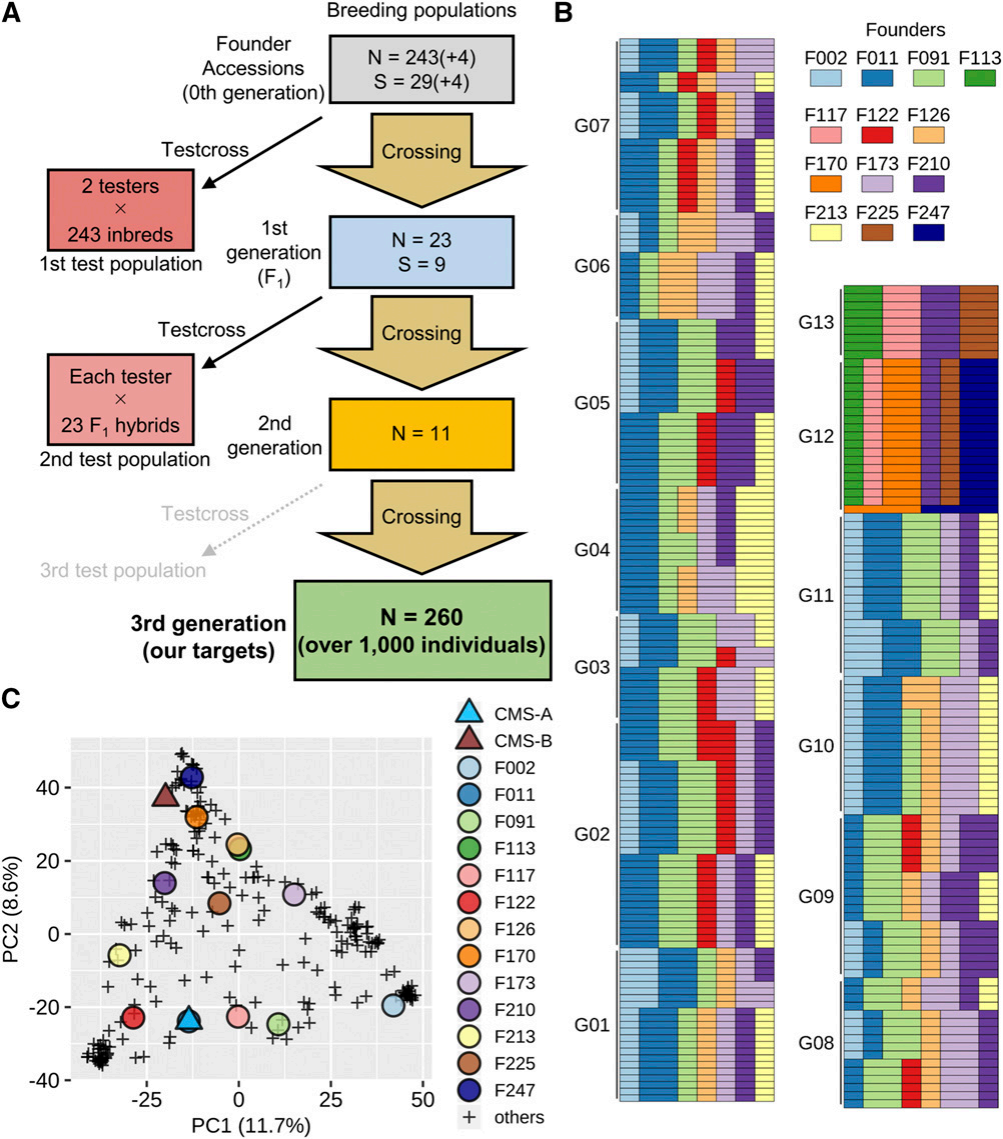
(A) The mating design for the sorghum breeding population. (B) The genetic proportion of 13 remaining founders in 260 breeding families (3^rd^ generation). The 260 families (each row) are divided into 13 groups (G01-G13) based on the proportion of eight progenitors (columns). (C) Principal component analysis on marker genotype (the two testers and base population). The testers (CMS-A and -B) and 13 remaining founders are shown in addition to the other base accessions.

The long-term goal of our project is the breeding of bioenergy sorghum. F_1_ hybrid cultivars for bioenergy sorghum require both high biomass and high sugar contents. The mating design used to derive the sorghum breeding population is described in Figure 1A. We use the term “family” as a full-sib family derived from the same parents, specifically for segregating generations. The term “individual” is used to describe each plant. The term “genotype” is used to identify genetically different plants. Genotype may refer to a single individual of a breeding family or a genetically uniform inbred accession or F_1_ hybrid.

The breeding population used in this study corresponds to the 3^rd^ intermating generation derived from the base population (the 0^th^ generation). To combine diverse genetic variations derived from the base population, intercrossing was performed in each generation. In theory, the maximum number of founders per family was eight in the 3^rd^ generation. For selection, at least one tester was crossed with each breeding candidate to check the progeny performance. In the selection, total weight (TW) and the Brix value of culm juice (BR) of testcrosses were considered as the main target traits, although other traits were also considered secondarily (see Phenotypic data). The selection criteria were determined comprehensively because the decision of the best testcross across multiple traits was generally difficult (*i.e.*, it depended on the breeder’s eye to some extent).

### Breeding populations

A base population (the 0^th^ generation) was composed of 243 inbred accessions, which had been obtained from public genebanks (Table S1). For the next population (the 1^st^ generation), we selected 29 accessions from the base population based on the progeny performance with each tester separately. Four accessions that were not tested in this project were added to the 29 accessions. Therefore, a total of 33 accessions was used as the parents for the 1^st^ generation. We performed 23 intercrosses among the 33 accessions, which corresponds to the 1^st^ generation (23 intra-population F_1_ hybrids). Of the 23 F_1_ hybrids, 9 F_1_ hybrids were selected for the next population (the 2^nd^ generation). All crosses genetically segregate starting in the 2^nd^ generation. The 2^nd^ generation included a total of 11 families, which were produced by 10 intercrosses among 8 F_1_ hybrids (S_0_ progenies) in addition to a family derived from the selfing of an F_1_ hybrid (S_1_ progenies). In this study, we used all 11 families of the 2^nd^ generation as the parents for the next population without selection. The next population (3^rd^) was derived from intercrosses among different families of the 2^nd^ generation. A few exceptions were derived from crosses within the same family (full-sib mating). A total of 137 individuals (average of 12.5 sibs per family in the 2^nd^ generation) was used as the parents for intercrossing, resulting in 260 full-sib families in the 3^rd^ generation.

Intercrossing was limited to the combinations within the group that had been selected for performance on each tester. This means that a selected genotype (or family) for a particular tester was intercrossed with another genotype (family) selected for performance in combination with the same tester.

### Test populations

Test populations were evaluated only for the selection of parents for the next breeding populations. In other words, test populations *per se* were not directly used as parents for the breeding populations. For selection, we performed testcrosses with two testers with cytoplasmic male sterility (CMS), CMS-A and CMS-B. The 1^st^ test population corresponds to the F_1_ hybrids between the two testers and the 0^th^ generation. Based on the performance of the F_1_ hybrids, the parental inbred accessions were selected for each tester.

The 2^nd^ test population corresponds to the three-way crosses between each tester and the 1^st^ generation. We created two sub-populations of selected F_1_s corresponding to the best families in combination with each of the two testers. Subsequent testcrossing on advanced generations involved the same tester used to initially select the breeding families. For example, when we selected multiple inbreds based on their testcross performance with the CMS-A tester in the 1^st^ test population, we evaluated the testcross between CMS-A and the F_1_ hybrid among the selected inbreds in the 2^nd^ test population. Because the 2^nd^ test population genetically segregates, the progeny rows for each testcross family were grown and evaluated. Considering the testcross performance (*e.g.*, the average and variance of phenotypic values within each testcross family, the suitability across target traits, and the degree of lodging and diseases), we applied the family selection to the 2^nd^ test population and selected families (*i.e.*, the parental F_1_ hybrids) for the next breeding population (the 2^nd^ generation).

### Phenotypic data

We performed a field trial of the S_0_ plants of the 3^rd^ generation *per se* (not as testcrosses) from June to September in 2017. The single experiment field was located at Corerepe, Sinaloa, Mexico (25° 37′ N, 108° 43′ W). We germinated seedlings in a greenhouse for three weeks before transplanting to the field. During the field trial, we adjusted the amount of irrigated water using drip irrigation. The fertilizer level was the standard for high-biomass sorghum (N:P:K = 17:60:50 kg ha^-1^). The space between ridges was 1 m with 15-cm distances between individuals in the same ridge. We divided the field into two blocks for allocating each breeding family. A total of 260 breeding families was randomly assigned to each plot across two blocks without replication. For check plots, the ancestral accessions that contributed to the 3^rd^ generation were incorporated into each block with at least a plot. In addition, 26 genotypes (F_1_ hybrids), which included 24 superior testcrosses generally selected from the 1^st^ test population and two high-biomass varieties provided by EARTHNOTE Co. Ltd., were replicated within blocks. Each plot included five individuals. For the breeding families, all five individuals within a plot were measured for the phenotypes, resulting in 1,300 individuals (260 families × 5 individuals). However, only 1,020 individuals (259 families, averagely 3.9 individuals per family) were used in this study due to missing data. For the ancestral accessions and checks, only two healthy individuals in a plot were measured. In this study, we evaluated six important traits for bioenergy sorghum (culm length [cm], CL; total biomass weight [kg transformed to natural logarithm], TW; the brix value of culm juice [%], BR; culm diameter [mm], CD; culm number [number in natural logarithm], CN; panicle length [cm], PL). All traits had been considered for each selection, mainly focusing on the performance of TW and BR.

To consider the field heterogeneity between the two experimental blocks, we calculated the adjusted phenotypic values using the following formula:

$$y_{ik}=\mu +g_{i}+b_{k}+e_{ik},$$

where $y_{ik}$ is the phenotypic value of the ith genotypes on the kth block, μ is an intercept, and $g_{i}$ is the effect of the ith genotype, which is treated as random for unreplicated individuals of the breeding population and as fixed for replicated ancestral accessions and checks (Kempton and Gleeson 1996), $b_{k}$ is the random effect of the kth block [where $b∼N(0,I\sigma_{b}^{2})$], and $e_{ik}$ is the residual [where $e∼N\left(0,I\sigma_{e}^{2}\right)$].

The adjusted phenotypic value of the ith genotype ($\tilde{y}$*_i_*) was calculated as ${\tilde{y}}_{i}=\hat{\mu }+{\hat{g}}_{i}$, where $\hat{\mu }$ is the estimated mean value and ${\hat{g}}_{i}$ is the best linear unbiased prediction of the ith genotype. The adjusted phenotypic values ($\tilde{y}$) were used as the response variable for the following Bayesian regression models. This model was implemented using the package RAINBOWR (Hamazaki and Iwata 2020) in R (R Core Team 2019).

### Marker data

DNA extraction and library preparation followed the procedure by Kobayashi *et al.* (2017). Although both the founder accessions and the breeding population were genotyped by restriction site-associated DNA sequencing (RAD-seq) (Baird *et al.* 2008), different restriction enzyme pairs (*Bgl*II and *Mse*I for the former, and *Bgl*II and *Eco*RI for the latter) were used due to a procedural reason. Therefore, we obtained different marker datasets for the founder accessions and the breeding population, respectively. We treated each marker dataset independently for the analysis.

Each marker dataset was available through the following procedures. We mapped the RAD reads to the sorghum reference genome sequence (*Sbicolor_313_v3.0*) (McCormick *et al.* 2018) using BWA version 0.7.15 (Li and Durbin 2009). We carried out variant calling using UnifiedGenotyper implemented in the Genome Analysis Toolkit version 3.5 (McKenna *et al.* 2010) and obtained the raw variant call format (VCF) output. Using VCF tools (Danecek *et al.* 2011), we chose only variant sites fulfilling the following conditions: mean depth range (3–30), missing score (≤5%), minor allele frequency (≥5%), quality value (>20), and bi-allelic single nucleotide polymorphism (excluding variant sites derived from insertions and deletions). Besides, only highly homozygous variant sites (the heterozygosity rate was less than 5%) were selected for the dataset of the founder inbred accessions. We imputed missing genotypes using Beagle 4.0 (Browning and Browning 2007). Of the highly linked variant sites (*r*^2^ ≥ 0.95 between two variant sites) on the same chromosome, only the first variant site in the VCF file (which was near the zero position on a chromosome) were kept. Finally, 6,410 (for the founder accessions) and 3,260 markers (the 3^rd^ breeding population) remained for the following analyses, respectively.

Principal component analysis (PCA) on each marker dataset was independently carried out using the function “prcomp” in R.

### Bayesian regression models

The Bayesian regression models used in this study can be classified into four categories: the additive linear model (A), the additive-dominance linear model (AD), the additive-dominance-epistasis linear model (ADE), and the Gaussian kernel model (GK). We adjusted marker genotype scores and calculated genomic relationship matrices using the natural and orthogonal interactions (NOIA) approach (Vitezica *et al.* 2017). The NOIA model is based on the genotypic frequency (not allele frequency), which can be applied also for populations without assuming a Hardy–Weinberg equilibrium, such as our breeding population. Here, we will briefly explain the statistical models.

The A model can be written as the next formula:

$$\begin{array}{c} {\tilde{y}}_{i}=\mu +\overset{L}{\underset{z=1}{\sum }}m_{iz}a_{z}+e_{i}, \end{array}$$ (1)

where ${\tilde{y}}_{i}$ is the adjusted phenotypic value of the ith genotype in the breeding population, μ is the mean value across all genotypes, L is the total number of markers, $m_{iz}$ is the marker coefficient of the ith genotype at the zth marker for the additive effect, $a_{z}$ is the additive effect of the zth marker, and $e_{i}$ is the residual [where $e∼N\left(0,I\sigma_{e}^{2}\right)$].

The AD model can be described as the extended form of the A model:

$$\begin{array}{c} {\tilde{y}}_{i}=\mu +\overset{L}{\underset{z=1}{\sum }}m_{iz}a_{z}+\overset{L}{\underset{z=1}{\sum }}m^{\prime }{}_{iz}d_{z}+e_{i}, \end{array}$$ (2)

where $m{’}_{iz}$ is the marker coefficient of the ith genotype at the zth marker for the dominance effect, and $d_{z}$ is the dominance effect of the zth marker.

The AD model can be further extended to the ADE model by incorporating the first-order epistasis terms:

$${\tilde{y}}_{i}=\mu +\overset{L}{\underset{z=1}{\sum }}m_{iz}a_{z}+\overset{L}{\underset{z=1}{\sum }}m{’}_{iz}d_{z}+\overset{L}{\underset{z=1}{\sum }}\overset{L}{\underset{w=1}{\sum }}(m_{iz}\cdot m_{iw})v^{aa}{}_{zw}+\overset{L}{\underset{z=1}{\sum }}\overset{L}{\underset{w=1}{\sum }}(m_{iz}\cdot m^{’}{}_{iw})v^{ad}{}_{zw}+\overset{L}{\underset{z=1}{\sum }}\overset{L}{\underset{w=1}{\sum }}(m^{’}{}_{iz}\cdot m^{’}{}_{iw})v^{dd}{}_{zw}+e_{i},$$ (3)

where $v^{aa}{}_{zw}$, $v^{ad}{}_{zw}$, and $v^{dd}{}_{zw}$ are the additive×additive, additive×dominance (including dominance×additive), and dominance×dominance epistatic effects between the zth and wth markers, respectively.

The epistatic effect terms are equivalent to the random effects that follow the multivariate Gaussian distributions, whose variance–covariance matrices are proportional to the Hadamard products of the corresponding relationship matrices (Jiang and Reif 2015). To derive epistatic matrices, the additive (A) and dominance (D) relationship matrices were first calculated using each marker coefficient based on the genotypic frequency (Vitezica *et al.* 2017). Using A and D, the additive×additive ($V^{aa}$), additive×dominance ($V^{ad}$, including dominance×additive), and dominance×dominance ($V^{dd}$) epistatic relationship matrices can be described as Vaa=A∘AtrA∘An,Vad=A∘DtrA∘Dn, and Vdd=D∘DtrD∘Dn, where X∘Y represents the Hadamard product of two matrices, X and Y, $tr\left(X\circ Y\right)$ is the trace, and n is the number of diagonal elements (*i.e.*, the number of genotypes). In this study, we fitted the ADE model using the epistatic relationship matrices ($V^{aa}$, $V^{ad}$, and $V^{dd}$) for the epistatic effect terms.

In the A, AD, and ADE models, four priors (BRR, BayesA, BayesB, and BayesC) were used to estimate a (additive marker effects) and d (dominance marker effects) (Meuwissen *et al.* 2001; Habier *et al.* 2011; Gianola 2013). In the AD and ADE models, the combination of the same priors for a and d was utilized (two different priors together were not examined).

Both additive and non-additive effects can be implicitly captured by the reproducing kernel Hilbert spaces (RKHS) regression based on a Gaussian kernel (GK) (Gianola and van Kaam 2008). The GK model can be written as ${\tilde{y}}_{i}=\mu +u_{i}+e_{i},$ where $u_{i}$ is the random effect of the ith individual [where $u∼N\left(0,K\sigma_{u}^{2}\right)$]. The Gaussian kernel is calculated as $K=\exp\left(-h\times S\right),$ where S is the squared-Euclidean distance matrix between genotypes in the breeding population, and h is the bandwidth parameter for adjusting the genetic covariance. To optimize the value of h in GP, we used the approach based on the restricted maximum-likelihood (REML) in each training dataset (Endelman 2011).

All Bayesian regression models were performed using Markov Chain Monte Carlo (MCMC) implementations in the R package BGLR (Pérez and de los Campos 2014). For the posterior density, the total iteration of the sampler is 30,000 and the number of discarded samples (as burn-in) is 15,000, which showed consistent results with more MCMC samples (300,000 iterations with 150,000 discards).

### Estimation of variance components

For estimating genetic variance components, we calculated genotypic values in each MCMC sample after burn-in (Lehermeier *et al.* 2017; Alves *et al.* 2019). The additive genotypic value (${\hat{g}}_{a}$) can be calculated using the following formula:

$${\hat{g}}_{ai}=\overset{L}{\underset{z=1}{\sum }}m_{iz}{\hat{a}}_{z},$$

where ${\hat{g}}_{ai}$ is the estimated additive value of the ith genotype, and ${\hat{a}}_{z}$ is the estimated additive effect of the zth marker. Similarly, the dominance genetic value (${\hat{g}}_{d}$) was also calculated in the AD and ADE models. The three epistatic genetic values (${\hat{g}}_{aa},{\hat{g}}_{ad},{\hat{g}}_{dd}$) were implicitly estimated in the ADE models. The total genetic value ($\hat{g}$) is the sum of these genetic values. The total genetic variance ($\sigma_{g}^{2}$) and variance components ($\sigma_{a}^{2},\sigma_{d}^{2},\sigma_{aa}^{2},\sigma_{ad}^{2},\sigma_{dd}^{2}$) were calculated as the variance of estimated values across all genotypes in each MCMC sample (Alves *et al.* 2019).

### Genome-wide association studies (GWAS)

For GWAS, we also used each MCMC sample after burn-in. In Bayesian whole-genome regression models, the estimated effect of any single marker can be small because of the correlation among adjacent markers. Fernando and Garrick (2013) applied the genomic window approach, which calculated the regional genetic variances using markers included in a genomic window. We calculated each regional genetic variance using a 1 Mb sliding window without overlaps. The formula for the regional additive variance in each MCMC sample can be as follows:

$${\hat{g}}_{a_{q}i}=\overset{P_{q}}{\underset{o=1}{\sum }}m_{io}{\hat{a}}_{o},$$

$${\hat{\sigma }}_{a_{q}}^{2}=\frac{1}{N}\overset{N}{\underset{i=1}{\sum }}{\left({\hat{g}}_{a_{q}i}-{\bar{g}}_{a_{q}}\right)}^{2},$$

where ${\hat{g}}_{a_{q}i}$ is the estimated additive genotypic value of the ith individual at the qth region, and $P_{q}$ is the number of markers included in the qth region. The relative additive variance at the qth region (${\hat{\zeta }}_{a_{q}}^{2}$) for the total genetic variance ${\hat{\sigma }}_{g}^{2}$ was estimated as follows:

$${\hat{\zeta }}_{a_{q}}^{2}=\frac{{\hat{\sigma }}_{a_{q}}^{2}}{{\hat{\sigma }}_{g}^{2}}.$$

The regional dominance variance can also be calculated using a similar procedure. Although any priors for marker effects can be used for GWAS, we described only the results of the ADE model with BayesB for the GWAS. In this study, we inferred regions with over 1% of the total genetic variance in each MCMC sample as being associated with target traits. The window posterior probability of association (WPPA) is calculated by counting the number of MCMC samples over the threshold for the total number of samples (Fernando and Garrick 2013).

For variance estimation and GWAS, Bayesian regression models were fitted as the full model using all genotypes of the breeding population.

### Genomic prediction

The predictive ability of the Bayesian regression models was evaluated by a fivefold cross-validation approach. We randomly divided the breeding population into five subsets. Of these five subsets, four subsets were used for training the model, and the remaining subset was validated using the trained model. This process was repeated until all subsets were validated, which corresponded to a single replication. The correlation coefficient (*r*) between the adjusted phenotypic values ($\tilde{y}$) and predicted values ($\hat{y}$) was recorded in each replication. We carried out the fivefold cross-validation approach for each model and each trait with 20 replications. After the values of the correlation coefficient were corrected using the Fisher *z*-transformation for model comparison, Tukey’s test (*P* < 0.01) was performed using the R package agricolae (de Mendiburu 2019).

### Data availability

RAD-seq data have been submitted to the NCBI Sequence Read Archive with the BioProject PRJNA614576. All supplemental materials are available at FigShare, including the phenotype, genotype, and ancestry data used in this study. Table S1 contains the results of GWAS. File S1 contains information on base accessions. File S2 contains phenotype data. File S3 contains genotype data. File S4 contains ancestry data. Other information is also available upon request. Supplemental material available at figshare: https://doi.org/10.25387/g3.12674369.

## Results

### Developing a sorghum breeding population

We developed a sorghum breeding population by the procedure summarized in Figure 1A. Of over 200 accessions in the base population (0^th^ generation), only 13 founders contributed to the ancestry of the 3^rd^ generation (Figure 1B). We classified 260 breeding families in the 3^rd^ generation into 13 groups (G01–G13) based on their relationships to the founders. The genetic contribution of each founder in the ancestry ranged from 12.5 to 25.0% in the breeding families, except a family in G12 in which F170 and F247 each contributed 50.0%. G12 and G13 had been selected based on the testcross with CMS-A while we had selected the other groups for CMS-B. Only one founder (F210) was included in the ancestry of both subpopulations selected on the basis of performance with each tester. The remaining 13 founders in the 3^rd^ generation represented a good sample of genetic variations of the base population (Figure 1C).

### Phenotypic variation of the breeding population

**Figure 2:**
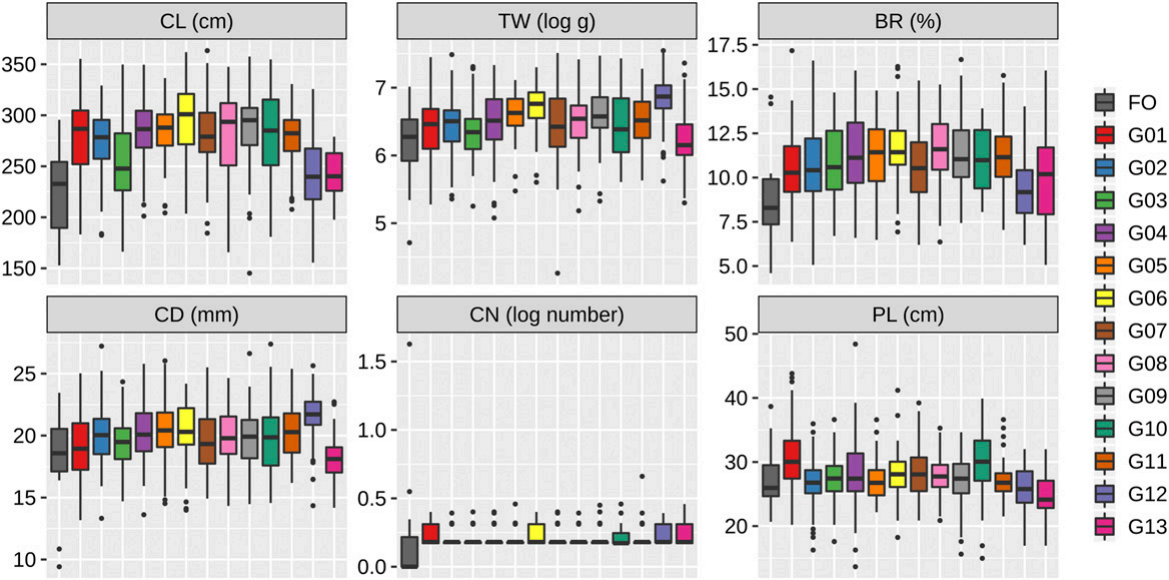
Variation among the adjusted phenotypic values. FO, founder accession; G0-G13, the 13 groups in the breeding population. CL, culm length; TW, total weight; BR, brix; CD, culm diameter; CN, culm number; PL, panicle length.

Compared to the founder accessions, each breeding group showed relatively high performances for most traits (Figure 2). The breeding groups, except for G13, had greater TW than did the founders. All groups also showed a higher performance than the founders in BR. Within the breeding population, the phenotypic variations were unique to each group. G12 showed the best performance in TW, which might be mainly due to the improvement of CD and CN. In G06, the improvement of all traits progressed simultaneously. G01 and G10 improved the performance of PL while the degree of the improvement was limited in the other groups.

### Genetic relationship within the breeding population

**Figure 3:**
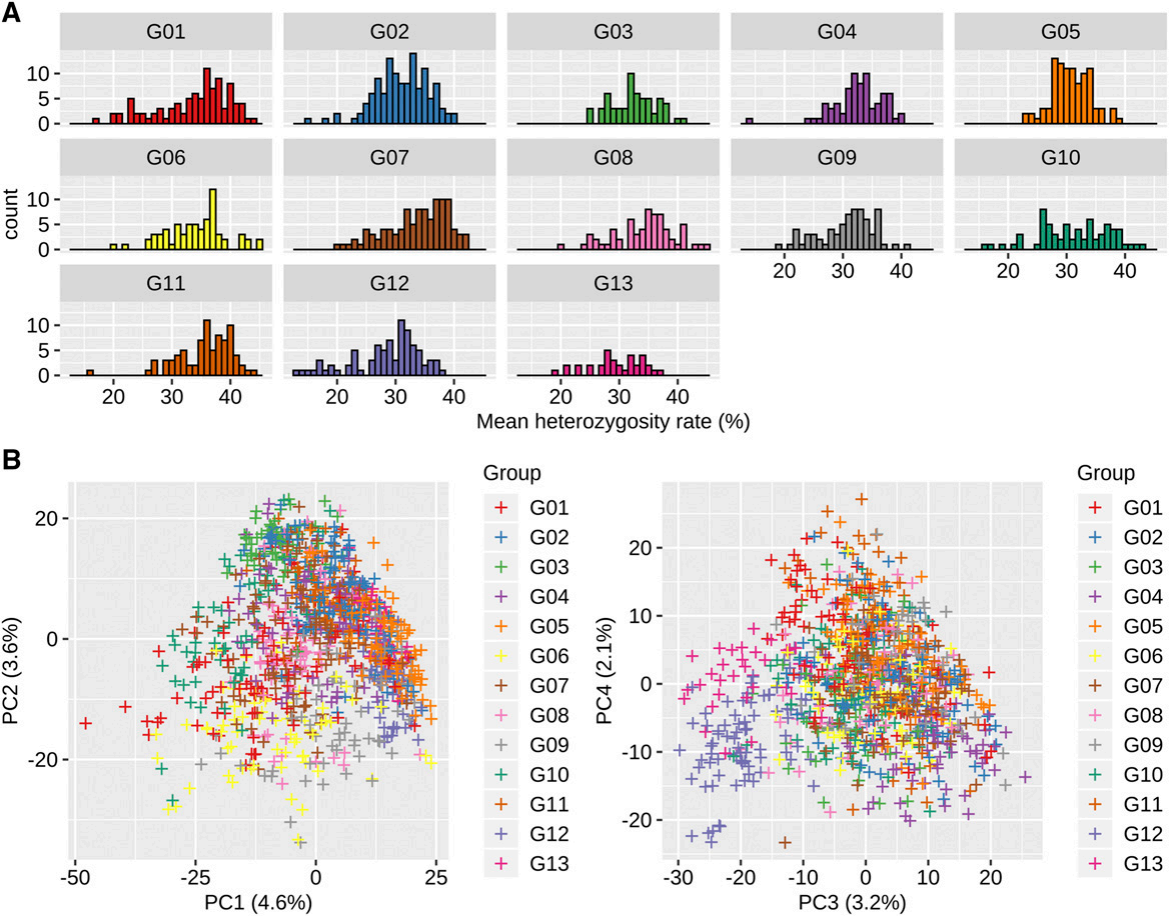
(A) Mean heterozygosity rate of marker genotype for the 13 groups in the breeding population. (B) Principal component analysis on marker genotype (the breeding population). G0-G13, the 13 groups in the breeding population.

The average heterozygosity of marker genotypes ranged from 20 to 40% among all groups (Figure 3A). The top two PCs of the marker genotypes of breeding candidates in the 3^rd^ generation showed continuous genetic variations without a distinct population structure (Figure 3B). Along in the third PC, G12 and G13 (which were selected on the basis of their performance when testcrossed to CMS-A) were partly separated from the other groups, indicating only a small amount of differentiation between the subgroups selected for each tester. These results might reflect the genetic composition, in which some founders were common to many breeding families (Figure 1B) and insufficient generations for genetic differentiation to occur.

### Genetic architecture of the breeding population

**Figure 4:**
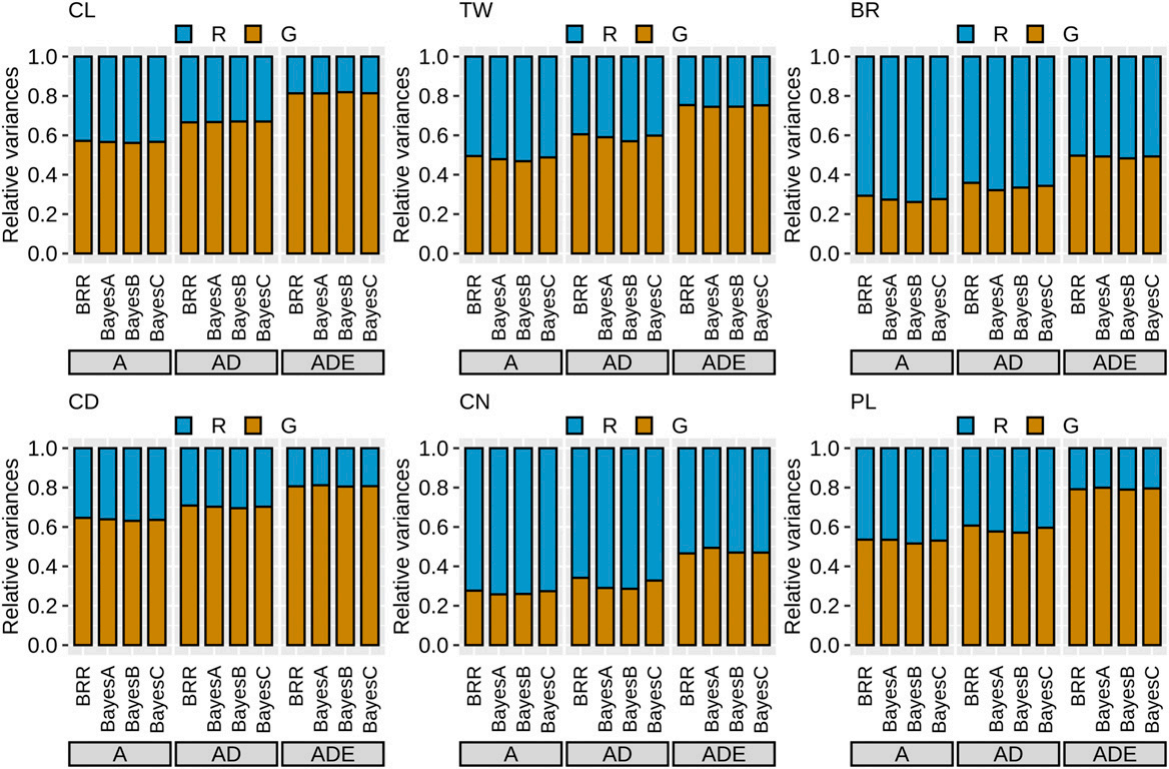
Genomic heritability in the sorghum breeding population. R, residual variance; G, genetic variance. CL, culm length; TW, total weight; BR, brix; CD, culm diameter; CN, culm number; PL, panicle length. A, additive models; AD, additive and dominance models; ADE, additive, dominance and epistasis models.

We estimated genomic heritability in the breeding population using Bayesian regression models (the A, AD, and ADE models), with different four priors for marker effects (Figure 4). In the A models, the genomic heritability of BR and CN was low (about 0.25), while the other traits (CL, TW, CD, and PL) showed intermediate values (about 0.50–0.65).

Compared to the A models, the AD models estimated the genetic variances at larger values in all traits although the residuals were relatively small. Furthermore, genomic heritability was greatest in the ADE models. The differences among the four priors for marker effects (BRR, BayesA, BayesB, and BayesC) were generally minor for the estimation of genomic heritability.

**Figure 5:**
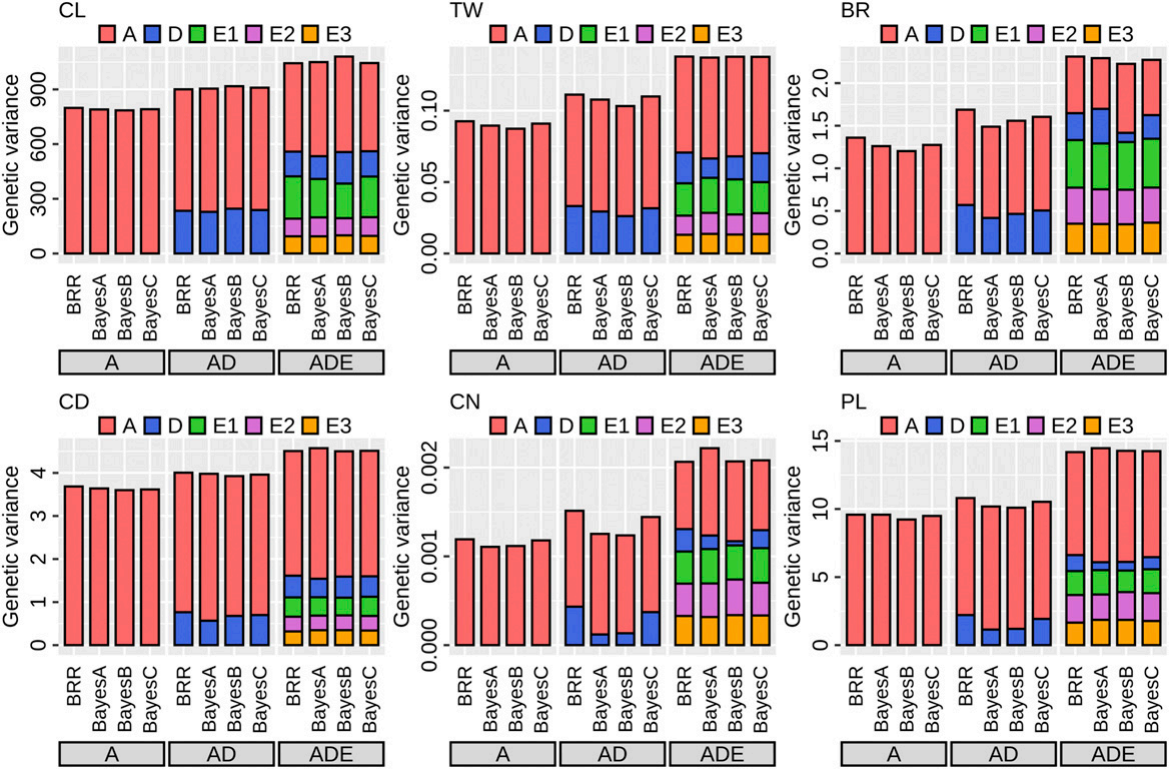
Estimation of genetic variance components. The additive (A), dominance (D), additive × additive (E1), additive × dominance (E2), and dominance × dominance epistatic (E3) variances were shown, respectively. CL, culm length; TW, total weight; BR, brix; CD, culm diameter; CN, culm number; PL, panicle length. A, additive models; AD, additive and dominance models; ADE, additive, dominance and epistasis models.

In addition to the total genetic variance, the variance components can be estimated in the AD and ADE models. In the AD models, the additive variance was larger than the dominance variance for all traits (Figure 5). The ratio of the dominance variance to the total genetic variance varied among traits, which was low in CN and PL, followed by the other traits. In the ADE models, the additive variance was the largest variance component, although the contribution of the additive variance was smaller than in the AD models. The three epistatic variances accounted for more than half of the total genetic variance in BR and CN, while epistatic variances contributed little to variation in CD. The priors for marker effects had only a limited influence on the estimation of the variance components in any trait.

### Genome-wide association studies

**Figure 6:**
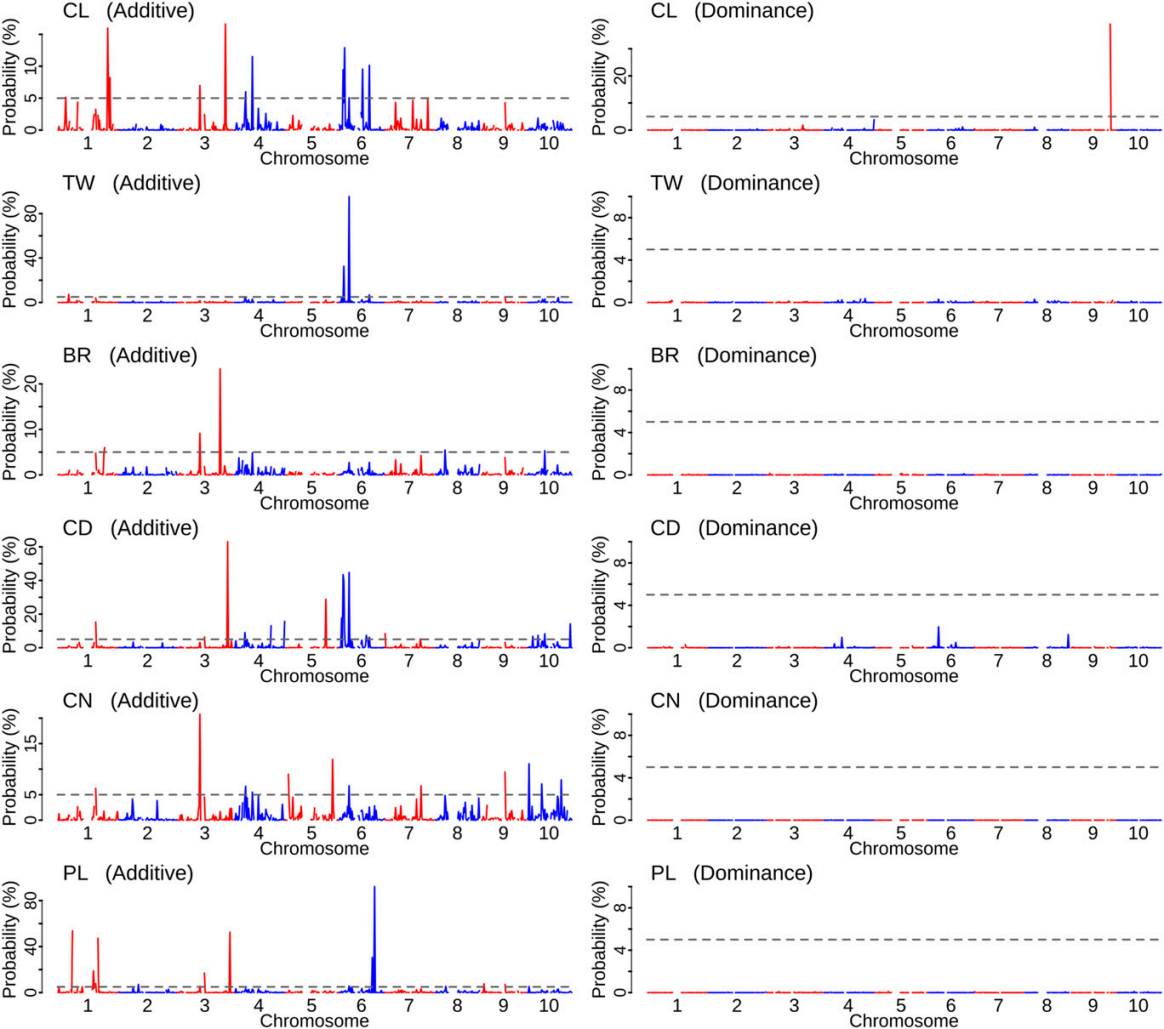
Genome-wide association studies for additive (A) and dominance (D) effects. In the ADE model with BayesB, the window posterior probability of association (WPPA) was calculated. Gray horizontal lines showed 5% of WPPA. CL, culm length; TW, total weight; BR, brix; CD, culm diameter; CN, culm number; PL, panicle length.

Using the ADE model with BayesB, we identified the regions associated with the additive and dominance effects (Figure 6). Except for CL, we detected no chromosomal regions with over 5% of WPPA for dominance effects. A region on chromosome 9 showed the association with the dominance effect in CL. In contrast, multiple regions showed associations with additive effects in all traits (Table S1). In CL, the strongest association with additive effects was located between 64–65 Mb on chromosome 3. In TW, a region on chromosome 6 (13–14 Mb) showed the association, in which the WPPA was over 95%. In BR, a region on chromosomes 3 had the highest WPPA. In CD, neighboring regions on chromosome 6 had high WPPA, in addition to multiple associations on other chromosomes. Also, multiple chromosomes showed probable associations with CN. In PL, two neighboring regions on chromosome 6 (44–45 and 47–48 Mb) had 30.5% and 92.1% of WPPA for additive effects, respectively.

### Genomic prediction

**Figure 7:**
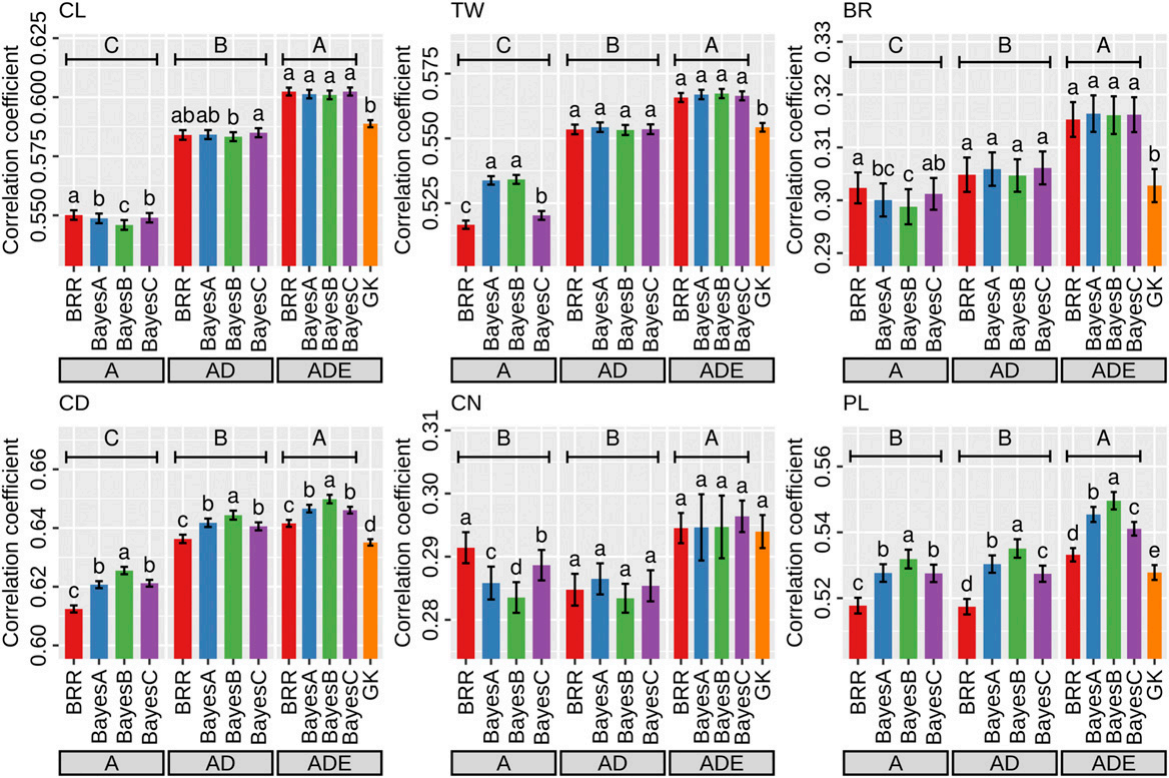
Genomic prediction based on the fivefold cross-validation. Error bars represent standard errors. Lowercase letters (a-d) above barplots indicate significant differences among marker priors in each model (the GK model was tested in the ADE models). Uppercase letters (A-C) above the lowercase letters indicate significant differences among models (the GK model was not included in the test). After the values of the correlation coefficient were corrected by Fisher *z*-transformation, both tests were performed by Tukey’s test (*P* < 0.01). CL, culm length; TW, total weight; BR, brix; CD, culm diameter; CN, culm number; PL, panicle length. A, additive models; AD, additive and dominance models; ADE, additive, dominance and epistasis models; GK, Gaussian kernel model.

We evaluated the prediction accuracy of the three linear models (A, AD, and ADE) and a non-linear model (GK) based on fivefold CV. The ADE models showed significant superiority to the A and AD models for all traits (Figure 7). Furthermore, The ADE models were superior to the GK model except for CN. The prediction accuracy of the GK model was similar to that of the AD models except for CN, while the AD models showed a higher prediction accuracy than the A models except for CN and PL. The A models were generally inferior to the other models although the differences among the models in prediction accuracy seemed to depend on traits to some degree (*e.g.*, the differences might be more distinct in CL than in the other traits). On the other hand, the influence of the priors for marker effects was relatively small across all predictions, although the difference among the priors seemed to depend on traits and models.

## Discussion

The first purpose of this study is to dissect the genetic architecture of the breeding population. The 3^rd^ generation population had improved trait performances *per se* compared to the founder accessions (Figure 2). Of particular interest was the possibility of important contribution of dominance (and dominance-related epistasis) to trait variation, because high heterozygosity remained in the early generation (Figure 3A). Our results showed that the additive variance was the main genetic variance component for all traits (Figure 5). In particular, over 50% of the total genetic variance for CD and PL was explained by the additive component across all AD and ADE models. The ratio of the dominance variance to the total genetic variance was generally limited in all traits, and no associations between marker dominance coefficients for specific genomic regions were detected except for CL (Figure 6).

Genetic architecture is not always consistent across different populations because of genetic sampling (Holland 2007). To overcome the differences due to genetic sampling, multi-parental populations have been used more recently, although dominance is not considered in these inbred populations (Buckler *et al.* 2009; Holland 2015). However, multi-parental mapping approaches are also affected by genetic sampling (Higgins *et al.* 2014). Our breeding population seems to maintain a large part of genetic diversity included in sorghum germplasm (Figure 1). Therefore, the genetic architectures characterized here may be relatively good indicators for what might be found in different populations in sorghum.

QTL detected previously using mapping populations might also contribute to our breeding population (Figure 6). A broad region on chromosome 6 contributing to CL, TW, and CD includes the *Ma1/Dw2* loci for maturation and height, which were cloned using mapping populations (Murphy *et al.* 2011; Hilley *et al.* 2017). Some QTL of the other traits might also correspond to identified loci using mapping populations, such as several associations with PL on chromosome 6 (Zou *et al.* 2012). In contrast, several major semi-dwarfism alleles, such as *dw1* on chromosome 9, seemed not to be segregating in the population. Although a region on chromosome 9 (52–53 Mb) showed a dominance association with CL, it was distant from the *Dw1* locus (Yamaguchi *et al.* 2016). It is possible that alleles contributing to semi-dwarfism might have been segregating in the base population, but quickly selected against during two selection cycles because they are unfavorable to high-biomass sorghums.

The second purpose of this study is the application of GP for evaluating early generations (Figure 7). Genomic heritability might be positively correlated with GP accuracy (Figure 4). In fact, genomic heritability can be an indicator for the implementation of GP (Guo *et al.* 2014). On the other hand, genomic heritability does not necessarily reflect the superiority of GP models because the balance between the goodness of fit and model complexity is important (Alves *et al.* 2019). The merit of a more complex model with non-additive effects may be attributed to the improvement of the prediction accuracy of breeding values and the genetic response (Varona *et al.* 2018). Furthermore, the utilization of genetic heritability needs to be carefully considered for inferring population parameters due to a sizable finite-sample bias (de los Campos *et al.* 2015).

The estimated genetic parameters can include the unreliability due to the correlations among variance components, particularly for epistatic terms (Vitezica *et al.* 2017). In fact, the total genetic variance was overestimated especially in the ADE models (Figures 4). The estimates of three epistatic variances seem to be generally similar to each other (*e.g.*, in CD), which might reflect their unreliability (Figure 5). Even if genetic architecture based on genetic variance components was dissected (Vitezica *et al.* 2018; Alves *et al.* 2019; Boeven *et al.* 2020), the importance of each genetic effect (*i.e.*, additive, dominance, and epistasis) cannot be inferred from the results (Huang and Mackay 2016).

The modeling of non-additive effects may also be important for the improvement of GP accuracy (Nishio and Satoh 2014; Jiang and Reif 2015; Varona *et al.* 2018), which was the third objective. For all target traits, the ADE models showed higher accuracies than the A and AD models (Figure 7). The result indicates that non-additive effects, especially epistatic terms, play a role for GP in the breeding population, even if the contribution of non-additive variances is different among traits (Figure 5). The merit of GP models considering non-additive effects is not clear in the literature (Varona *et al.* 2018). First, more complex models might be quite sensitive to training dataset size in empirical studies. Zhao *et al.* (2013) suggested that the large population size was necessary for the estimation of the dominance effect. Second, the genetic architecture might be different among traits, populations, and species. The underlying genetic architecture can directly affect the superiority of GP models (Howard *et al.* 2014). Unfortunately, the relative magnitudes of variance components do not reflect the functional importance (Huang and Mackay 2016). Nevertheless, the merit of modeling non-additive effects is related to the size of these variances to some extent (Alves *et al.* 2019).

Although additive genetic variance is important for the improvement of *per se* performance, GP models with non-additive effects may be useful to accurately predict the additive genetic value of a genotype which is affected by both additive and non-additive effects. In particular, when a breeding population is an early generation as the population evaluated in this study, dominant variations may not be negligible in the total genetic variations. To clarify the practical importance of non-additive variations, other potential usefulness of non-additive GP models trained on *per se* performance should necessarily be validated in future studies.

The GK model is an alternative method for considering non-additive effects (Gianola and van Kaam 2008). In this study, the GK model showed a lower predictive ability than the ADE models, except for CN (Figure 7). The advantage of the GK model over linear models may be influenced by the underlying genetic architecture (Howard *et al.* 2014). In fact, the GK model was not necessarily better than a linear model for the prediction of single-cross performance in maize (Kadam and Lorenz 2019). Further, the optimization of the hyperparameter h is necessary for the GK model (Endelman 2011). These results show that the ADE models have an advantage over the other models when non-additive effects need to be considered.

Gianola (2013) explained the property of various priors for marker effects in Bayesian regression models. Our results showed that the influences of the four priors (BRR, BayesA, BayesB, and BayesC) were generally small, although the underlying genetic architecture seemed to be different among traits (Figures 5, 6, and 7). In empirical analyses, the differences among priors may be smaller than expected from simulation studies (de los Campos *et al.* 2013). Because of no universally best priors (or models), several priors for marker effects should be examined for each trait (Momen *et al.* 2018).

Although we could give light on the genetic architecture of early generations in a sorghum breeding program, the genetic architecture of a population is different in every generation with the change of the genotypic frequency (Walsh and Lynch 2018). Further, the reduction of genetic variance generally progresses with selection (Bulmer 1976). The loss of linkage disequilibrium between markers and QTL also arises in advanced generations (Jannink 2010; Toro and Varona 2010). Therefore, the re-evaluation of the breeding population may be necessary after a few selection cycles (Iwata *et al.* 2011; Yabe *et al.* 2017).

The limitations of epistasis for selection might be another issue. For mate allocation, recombination fractions across the genome need to be considered (Varona *et al.* 2018). Even if epistasis contributes to genetic variance, most epistatic variances can be incorporated into the additive variance with the changes of the genotypic frequency under a finite effective population size (Walsh and Lynch 2018). To exploit epistasis in plant breeding, new methods to overcome the limitations of models and breeding strategies based on additive variance alone are necessary (Holland 2001). On the other hand, the role of epistasis is recently reconsidered for the long-term response to selection (Paixão and Barton 2016). Several strategies based on the additive model are proposed for long-term genetic gain (De Beukelaer *et al.* 2017; Gorjanc *et al.* 2018; Allier *et al.* 2019). Non-additive models may be considered in future studies if they contribute to the enhancement of the genetic response through more accurate estimation of additive breeding values (Varona *et al.* 2018).
